# Complementary and alternative medicine recommendations for depression: a systematic review and assessment of clinical practice guidelines

**DOI:** 10.1186/s12906-020-03085-1

**Published:** 2020-10-07

**Authors:** Jeremy Y. Ng, Zainib Nazir, Hayley Nault

**Affiliations:** Department of Health Research Methods, Evidence, and Impact, Faculty of Health Sciences, McMaster University, MDCL-2112, 1280 Main Street West, Hamilton, Ontario L8S 4K1 Canada

**Keywords:** depression, complementary and alternative medicine, systematic review, AGREE II, clinical practice guideline

## Abstract

**Background:**

Up to 50% of individuals diagnosed with depression are known to use complementary and alternative medicine (CAM). The aim of this study was to identify the quantity and assess the quality of CAM recommendations in clinical practice guidelines for the treatment and/or management of depression in adults using the Appraisal of Guidelines, Research and Evaluation II (AGREE II) instrument.

**Methods:**

A systematic review was conducted to identify depression guidelines. MEDLINE, EMBASE and CINAHL were searched from 2008 to 2018. The Guidelines International Network and the National Center for Complementary and Integrative Health websites were also searched. Eligible guidelines published by non-profit agencies on treatment of depression for adults were assessed with the AGREE II instrument twice, once for the overall guideline and once for the CAM sections.

**Results:**

From 931 unique search results, 19 guidelines mentioned CAM, of which 16 made CAM recommendations. Scaled domain percentages from highest to lowest were as follows (overall, CAM section): clarity of presentation (87.0, 66.1%), scope and purpose (80.9, 77.6%), stakeholder involvement (62.0, 44.3%), editorial independence (61.6, 61.6%), rigour of development (58.0, 52.0%), and applicability (42.2, 25.4%). Quality varied within and across guidelines. Only 1 of 16 guidelines was recommended without modifications for both its overall and CAM sections by both appraisers.

**Conclusions:**

There are multiple depression guidelines containing CAM recommendations available and there are a comprehensive set of CAM therapy options for depression. The quality of guidelines varied within and across guidelines and the quality of CAM recommendations was generally lower than the overall recommendations in the guidelines for all domains except editorial independence. Generally, characteristics of guidelines, including the year of publication and region of development varied across the guidelines irrespective of quality. Guidelines with higher AGREE II scores can serve as a guide to facilitate communication between patients and medical professionals regarding CAM use for depression, while guidelines with lower scores could be improved in future updates using the AGREE II instrument as a guide.

## Background

Depression is a common mood disorder that is becoming increasingly prevalent, affecting over 264 million people worldwide [1]. It is one of the leading causes of disability or short-term and long-term health loss among adults in the world [2] and it impairs a person’s ability to perform normal activities of daily life including sleeping, eating or working [3, 4]. Depression is classified based on the criteria outlined in the Diagnostic and Statistical Manual of Mental Disorders (DSM) or the International Classification of Diseases (ICD) [4, 5]. It is defined by the intensity and persistence of symptoms such as a reduction in mood, hopelessness, fatigue, a loss of interest in almost all activities, appetite or weight changes, and a decrease in activity, manifesting close to every day for a minimum of 2 weeks, where the depressive episodes are not associated with or a result of another medical condition or a substance or drug [3, 5]. Various methods are used to diagnose depression including a patient interview, and symptom scales or questionnaires such as the Patient-Health Questionnaire (PHQ), Hamilton Depression Rating Scale (HDRS), or Inventory for Depressive Symptomatology (IDS) [6]. In 2017, according to the National Survey of Drug Use and Health, approximately 17.3 million adults experienced depression in the United States (US) [7]. Several studies have shown that the prevalence of complementary and alternative medicine (CAM) use in patients with depression is high and that those with mental health disorders, specifically depression, are more likely to seek and use CAM therapies than those with other health conditions [8–15]. In a nationally representative survey of the US, it was found that about 53.6% of respondents with severe depression accessed CAM for treatment within the last year [14], and in a prior survey the prevalence was 40% [13]. According to the National Center of Complementary and Integrative Health (NCCIH), CAM is a diverse set of therapeutic forms or “health care approaches that are not typically part of conventional medical care or that may have origins outside of usual Western practice” [16]. Within this classification, NCCIH further differentiates complementary from alternative approaches; complementary approaches are used as an adjunct to conventional approaches while alternative approaches are used as a substitute to conventional ones [16].

There are over 120 CAM therapies that purportedly help treat depression or relieve depressive symptoms [12], the most common of which are herbal remedies including St. John’s Wort (*Hypercium perforatum*), S-adenosyl-L-methionine (SAMe); vitamins, minerals or dietary supplements including folate or omega-3 fatty acids; physical or mind-body therapies such as light therapy, acupuncture and exercise; and mindfulness psychotherapies including mindfulness-based cognitive behavioural therapy (MCBT) [8, 12, 15, 17]. Despite the increasing demand for CAM, there is a general lack of CAM training and education for medical professionals [18–20]. Furthermore, many mental health practitioners have a general knowledge gap and may feel uncomfortable discussing CAM therapies, therefore, hindering them from communicating or extending evidence-based CAM recommendations to patients [21]. Thus, there is a need for reliable evidence-based resources for healthcare professionals to increase discussion regarding the use of CAM in the treatment of depression with patients.

The Institute of Medicine (IOM) defines clinical practice guidelines as “statements that include recommendations intended to optimize patient care that are informed by a systematic review of evidence and an assessment of the benefits and harms of alternative care options” [22]. Moreover, evidence-based guidelines contain therapy recommendations that are typically relied upon by healthcare professionals to understand their risks and benefits, and they thereby lend to informed and shared decision-making with patients [23, 24]. To our knowledge, there are only three studies in the literature that have previously assessed the quality of depression guidelines in general with different scopes and methods [25–27]; one study assessed the overall guidelines for depression in adults [25], one study assessed the guidelines for the management of depression in primary care in general [26], and one study assessed guidelines for both depression and anxiety in children and youth [27]. No studies have previously assessed the CAM recommendations in guidelines for the treatment and/or management of depression in adults. Nonetheless, depression remains a condition where the prevalence of CAM use across patients is high [8–15]. An evaluation of the quality of CAM sections in depression guidelines could provide a greater insight into gaps in the literature and healthcare resources, as well as indicate points of improvement to guide and facilitate future research, the development of higher quality guidelines and thus more evidence-based CAM recommendations. Therefore, the purpose of this study was to identify the quantity of CAM mention and recommendations in guidelines for the treatment and/or management of depression and assess their quality using the AGREE II instrument.

## Methods

### Approach

A systematic review was performed to identify guidelines for the treatment and/or management of depression following standard methods [28] and the Preferred Reporting Items for Systematic Reviews and Meta-Analyses (PRISMA) criteria [29]. A protocol was registered with PROSPERO; registered number: CRD42019132295. Eligible guidelines that provided CAM recommendations were evaluated using the well-accepted and validated Appraisal of Guidelines, Research and Evaluation II (AGREE II) tool [30] twice, once for the overall guideline and a second time for the CAM sections of the guideline. The AGREE II instrument is comprised of 23 items organized within six domains: scope and purpose, stakeholder involvement, rigor of development, clarity and presentation, applicability, and editorial independence.

### Eligibility criteria

The Population, Intervention, Comparison and Outcomes (PICO) framework was used to define the eligibility criteria for the depression guidelines. Eligible *populations* were adults aged 18+ years with unipolar or major depression. Guidelines that focused solely on geriatric populations however were excluded. In terms of *interventions*, only guidelines that focused on the treatment and/or management of depression were deemed eligible for the purpose of identifying whether any CAM therapies were mentioned or recommended. With respect to *Comparisons,* we aimed to assess the quality of depression guidelines, specifically commenting on CAM sections. In other words, we aimed to determine the proportion of depression guidelines that made CAM recommendations and the quality by which they did so. Outcomes included AGREE II scores to assess of the quality of the content and format of the overall and CAM sections of the guidelines. Eligible guideline were in the English language, accessible to the public or via the McMaster library system, and developed by non-profit organizations including academic institutions, government agencies, disease-specific foundations, specialty/expert groups, international organizations or professional associations or societies. This timeframe provided developers with a minimum of five years since the publication of AGREE II and thus time to apply the tool which outlines methods for the generation of high-quality guidelines. Publications that were not evidence-based clinical practice guidelines focusing on the treatment and/or management of depression were deemed ineligible. For example, consensus statements, protocols, abstracts, conference proceedings, letters or editorials, studies based on primary studies that evaluated depression management or treatment or studies that focused on depression curriculum, education, training, research, professional certification or performance were not eligible. It is important to note that the AGREE II tool was used to assess only those eligible guidelines that provided CAM therapy recommendations. Thus, depression guidelines that had no CAM mention or only mentioned CAMs without making any CAM recommendations were not assessed using AGREE II. This allowed for the comparison between AGREE II scores of the overall guideline and specifically the CAM sections. Only demographic information was collected and described for the eligible guidelines that only made mention of CAM therapies and did not make any CAM therapy recommendations.

### Searching and screening

On October 11, 2018, a search of three databases, MEDLINE, EMBASE, and CINAHL, was conducted from 2008 to October 9, 2018 inclusive. A sample search strategy is provided in Supplementary File 1. In addition, the Guidelines International Network (G-I-N), a website containing a library or repository of guidelines [31] was searched with keywords reflective of the eligibility criteria of this study, including “depression”, “major depressive disorder”, and “depressive disorder”. Finally, the NCCIH website was also searched as it provided a single list of CAM guidelines [https://nccih.nih.gov/health/providers/clinicalpractice.htm]. To identify the eligible sources, a two-step screening process was conducted by ZN and HN individually; they each screened the titles and abstracts of all sources, after which they each screened the full texts of the sources. Each screening step was standardized by JYN who helped to resolve discrepancies in the selection of eligible sources between ZN and HN.

### Data extraction and analysis

The following data were extracted and summarized from each of the eligible guidelines: date of publication; country of first author; type of organization that published the guideline (academic institutions, government agencies, disease-specific foundations, specialty/expert bodies or groups, international organizations, or professional associations or societies); and if there was any mention of CAM. Moreover, the following data were also extracted: the specific CAM therapies that were mentioned if present; recommendations of CAM therapies if present; CAM funding sources; and whether any CAM providers were members of the guideline development group or panel. Associated tools and/or resources to support the implementation of the guidelines were sought from each guideline’s developer website. Data from these resources were used to assess applicability. Some CAM therapies mentioned in the eligible guidelines of this study were not widely known as or as easily defined as either standard non-CAM or CAM treatments by ZN and the other researchers, so the literature was searched to seek an understanding of each therapy and determine it as either CAM or non-CAM [12, 17, 32–50], while consulting the NCCIH and literature that has previously provided an operational definition of CAM [16, 51].

### Guideline quality assessment

The quality of the eligible guidelines was assessed using the AGREE II instrument following standardized methods described in the user manual [30]. All 23 items within the 6 domains of the AGREE II tool were scored on a 7-point Likert scale, where “1” reflects “strongly disagree” and “7” reflects “strongly agree”. Next, the quality of the overall guideline was rated on the same scoring system for an overall assessment score and then it was determined if the guideline should be recommended for use or not. Initially, a pilot test was performed in which all three authors applied the AGREE II tool to appraise three separate guidelines, after which inconsistencies in scores were discussed and resolved to standardize scoring. The AGREE II tool was then independently applied to the eligible guidelines of this study by ZN and HN. Those guidelines with CAM recommendations were assessed using the AGREE II tool twice to assess the overall guideline and then to assess the CAM sections specifically. The AGREE II questions were modified for application to the CAM sections of the guidelines specifically, as described in Supplementary File 2. Discrepancies between the scores of ZN and HN were discussed and resolved in consultation with JYN, without unduly modifying scores. Next, the average appraisal scores, average overall assessments, and scaled domain percentages of the guidelines were generated. Each appraiser’s average of all 23 scored items for each guideline was calculated separately and those two values were then averaged to generate the average appraisal score of each guideline. The overall assessment scores of both appraisers were averaged for each guideline to generate the average overall assessments. Scaled percentages were calculated for each domain to allow for comparisons between the different aspects of each guideline. The scaled domain percentage was calculated for each of the six domains by taking the sum of the individual scores of the two appraisers within the domain, scaling it to the minimum and maximum possible score for that domain, and then transforming it into a percentage. For each domain, the average of the guidelines’ scaled domain percentages was also generated.

## Results

### Search results (Fig. 1)

Searches retrieved 1043 total items, 931 were unique, and 863 titles and abstracts were eliminated, leaving 68 full-text guidelines that were considered. Of those, 49 were not eligible, because they were not clinical practice guidelines (*n* = 16), they were not focused on depression (n = 1), they addressed depression associated with another condition or disease (*n* = 5), there was a more recent version of the guideline available (*n* = 4), they were not in English (*n* = 9), they were published prior to the eligible timeframe (n = 5), they did not focus on the target population (*n* = 7), they were not treatment focused (n = 1), or they were withdrawn/invalid guidelines (n = 1), leaving 19 guidelines eligible for review [52–70]. Thus, a total of 19 guidelines were eligible, of which 1 mentioned CAM but did not make any CAM recommendations [68], and 2 made no mention of CAM [69, 70]. The remaining 16 guidelines made CAM therapy recommendations; these guidelines were assessed using the AGREE II tool.

**Fig. 1:**
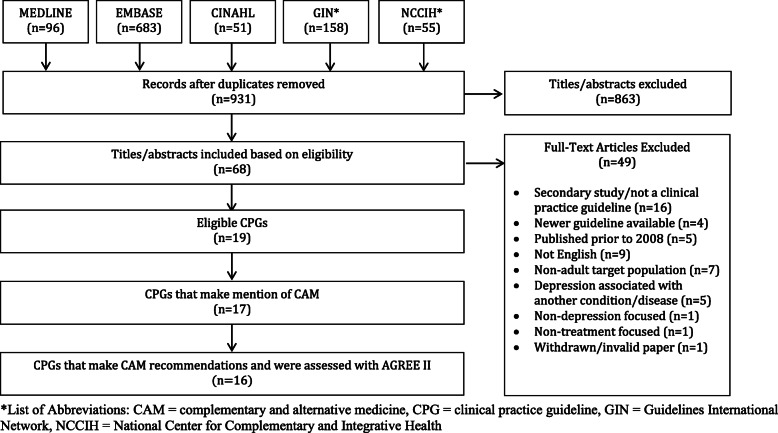
PRISMA Diagram

### Guideline characteristics

Eligible guidelines were published from 2008 to 2018 in the USA (*n* = 5), Canada (*n* = 4), Germany (*n* = 3), the UK (*n* = 2), Australia (*n* = 1), China (*n* = 1), Poland (*n* = 1), Scotland (*n* = 1), and Spain (*n* = 1) [52–70]. The guidelines were funded and/or developed by professional associations or societies (*n* = 5), academic institutions (*n* = 2), international organizations (*n* = 2), specialty/expert groups (*n* = 7), a combination of a specialty/expert body and a government agency (*n* = 1), a combination of a specialty/expert group and an international organization (*n* = 1), and a specialty/expert group and an academic body (*n* = 1).

**Fig. 2:**
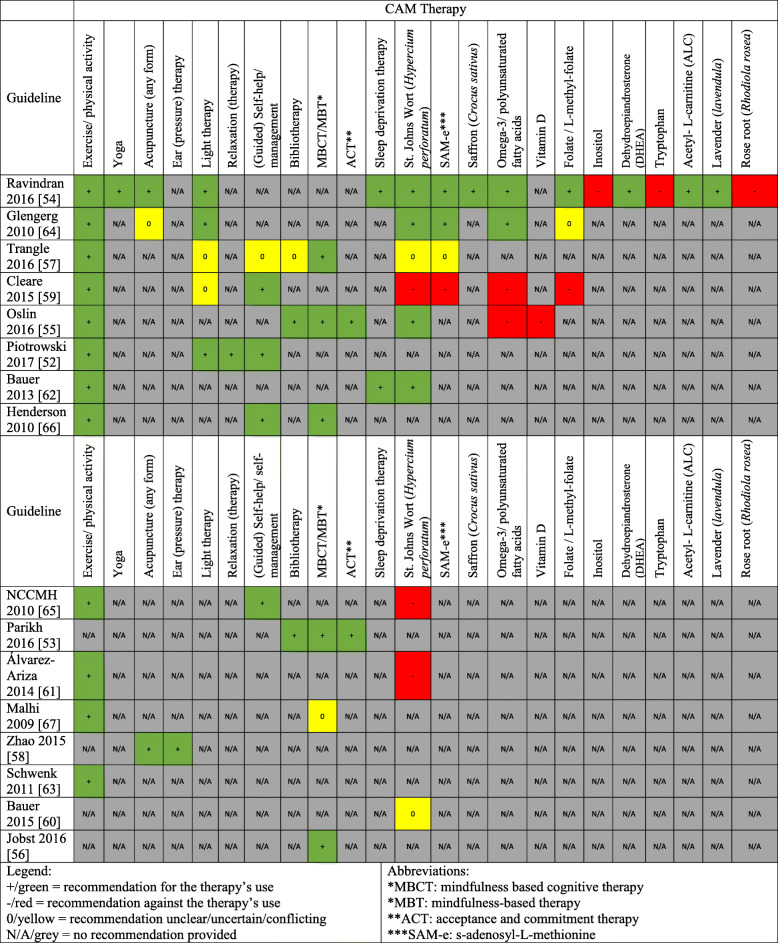
Summary of CAM Recommendations in Clinical Practice Guidelines

CAM recommendations were made in 16 guidelines [52–67] and included the following therapies: St. John’s Wort (*Hypercium perforatum*) (*n* = 9), SAMe (S-adenosyl-L-methionine) (*n* = 4), exercise/physical activity (*n* = 12), yoga (*n* = 1), acupuncture (*n* = 3), specialized forms of acupuncture including electro-acupuncture, scalp acupuncture, filiform needle acupuncture, body acupuncture involving regulating mind and soothing the ‘Gan’, scalp electro-acupuncture (*n* = 1), ear (pressure) therapy/ pressing of ear points with herbal seeds (*n* = 1), relaxation training/therapy (*n* = 1), (bright) light therapy (*n* = 5), (guided) self-help/self-management (*n* = 5), bibliotherapy (*n* = 3), mindfulness based therapy/mindfulness based cognitive therapy (MBT/MCBT) (*n* = 6), acceptance and commitment therapy (ACT) (*n* = 2), sleep deprivation/wake therapy (*n* = 2), saffron (*Crocus sativus*) (*n* = 1), omega-3/ polyunsaturated fatty acids (*n* = 4), vitamin D (*n* = 1), folate/L-methylfolate (*n* = 3), inositol (*n* = 1), dehydroepiandrosterone (DHEA) (*n* = 1), tryptophan (*n* = 1), acetyl- L-carnitine (ALC) (*n* = 1), lavender (*Lavendula*) (*n* = 1), and rose root (*Rhodiola rosea*) (*n* = 1). The aforementioned characteristics of these 16 guidelines are described in Table 1 and a summary of the CAM recommendations in these eligible guidelines are presented in Fig. 2. One of the 16 guidelines had a CAM funding source, and 5 guidelines included active or retired CAM providers as part of the guideline development group.

**Table 1:** Characteristics of Eligible Guidelines

Guideline	Country (First Author)	Developer	CAM category	Guideline topic
Piotrowski 2017 [52]	Poland	Polish Psychiatric Association – Wroclaw Division, the Polish Society of Family Medicine and the College of Family Physicians in Poland	General CAM	Diagnosis and treatment of depressive disorders in primary health care
Parikh 2016 [53]	Canada	Canadian Network for Mood and Anxiety Treatments (CANMAT)	Psychotherapeutic CAM	Psychological treatments for the management of adults with major depressive disorder
Ravindran 2016 [54]	Canada	Canadian Network for Mood and Anxiety Treatments (CANMAT)	General CAM	Complementary and alternative medicine treatments for the management of adults with major depressive disorder
Oslin 2016 [55]	United States	The Management of Major Depressive Disorder Working Group with support from The Office of Quality, Safety and Value and Office of Evidence Based Practice - USA Army Medical Command; US Department of Defense and Department of Veterans Affair (VA/DoD)	General CAM	The management of major depressive disorder
Jobst 2016 [56]	Germany	European Psychiatric Association	Psychotherapeutic CAM	Psychotherapy in chronic depression across Europe
Trangle 2016 [57]	United States	Institute for Clinical Systems Improvement Inc	General CAM	Adult depression in primary care
Zhao 2015 [58]	China	Depression Guideline Development Group, Sponsored by Traditional Medicine Office, Western Pacific Region, World Health Organization	Acupuncture	Acupuncture to treat depression
Cleare 2015 [59]	United Kingdom	British Association for Psychopharmacology	General CAM	Treating depressive disorders with antidepressants
Bauer 2015 [60]	Germany	World Federation of Societies of Biological Psychiatry (WFSBP)	General CAM	Biological treatment of unipolar depressive disorders: Maintenance treatment of major depressive disorder
Álvarez-Ariza 2014 [61]	Spain	Ministry of Health, Social Services and Equality; Galician Agency for Health Technology Assessment	General CAM	Management of depression in adults
Bauer 2013 [62]	Germany	World Federation of Societies of Biological Psychiatry (WFSBP)	General CAM	Biological treatment of unipolar depressive disorders: The acute and continuation treatment of unipolar depressive disorders
Schwenk 2011 [63]	United States	University of Michigan (Quality Management Program)	General CAM	Treatment of depression
Gelenberg 2010 [64]	United States	American Psychiatric Association	General CAM	Treatment of patients with major depressive disorder
NCCMH 2010 [65]	United Kingdom	National Collaborating Centre for Mental Health (NCCMH), commissioned by the National institute for Health & Clinical Excellence (NICE)	General CAM	The treatment and management of depression in adults
Henderson 2010 [66]	Scottland	Scottish Intercollegiate Guidelines Network (SIGN)	General CAM	Non-pharmaceutical management of depression in adults
Malhi 2009 [67]	Australia	Unclear; This paper was part of a larger project, Mood Matters, which represented a collaboration between the Northern Sydney Central Coast Mental Health Drug & Alcohol (NSCCMHDA), NSW Health Clinical Redesign Program and the CADE Clinic, University of Sydney	General CAM	Management of depression in adults

### Guidelines mentioning CAM without recommendations

There was one guideline that only made mention of CAM without making any CAM recommendations [68]. Regarding the one psycho-therapeutic CAM mentioned, which was acceptance and commitment therapy (ACT), only a brief statement was made indicating that this is an available treatment option for depression without any comment on supporting evidence. A detailed discussion comparable to the non-CAM treatments in this guideline was made for acupuncture, exercise, and St. John’s Wort. A detailed description of the adverse effects and risks/side effects of St. John’s Wort was specifically discussed as well. The remaining CAM therapies, including omega-3 fatty acids, SAMe, fatty acids, yoga, and meditation, were vaguely mentioned with brief statements on the quality of evidence relative to the non-CAM treatments which were discussed in more detail.

### Average appraisal scores and average overall assessments regarding use of guidelines: overall guideline

The average appraisal scores and average overall assessments regarding use for each of the 16 guidelines with CAM recommendations are presented in Supplementary File 3. From the overall average appraisal scores of each of the guidelines, the lowest score was 3.4 while the highest was 6.0, using the seven-point Likert scale (where “7” reflects strongly agree that the criteria for that item is fulfilled); only 1 guideline received an average appraisal score lower than 4.0, 10 guidelines received an average appraisal score greater than 4.5, and 5 guidelines received an average appraisal score greater than 5.0. Meanwhile, the lowest average overall assessment score was 3.3, while the highest was 6.0, of the 16 guidelines that were assessed; only 1 guideline had an average overall assessment score lower than 4.0, 11 guidelines achieved a score of 4.5 or greater, and 5 guidelines achieved a score of 5.0 or greater.

### Average appraisal scores and average overall assessments regarding use of guidelines: CAM sections

The average appraisal scores and average overall assessments regarding use for each of the 16 guidelines with CAM recommendations are shown in Supplementary File 3. From the average appraisal scores for the CAM sections of each of the 16 guidelines, the lowest score was 1.9 and the highest score was 5.8 on the seven-point Likert scale; 3 guidelines received an average appraisal score lower than 3.0, 11 guidelines received an average appraisal score of 4.0 or greater, and 2 guidelines received an average appraisal score greater than 5.0. For the CAM average overall assessments of the 16 guidelines, the lowest score was 2.0, while the highest was 6.0; 4 guidelines achieved an average overall assessment score of 3.0 or lower, 11 guidelines achieved a score of 4.0 or greater, and 2 guidelines achieved a score of 5.0 or greater.

### Overall recommendations: overall guideline (Table 2)

The two appraisers only recommended 1 of the 16 guidelines as Yes without modifications [65]. For 13 of the 16 guidelines, both appraisers concurred in their decision regarding the overall recommendation; one guideline was rated as No by both appraisers [52], and 11 were rated as Yes with modifications by both appraisers [53, 54, 56, 58–63, 66, 67]. Finally, 3 guidelines were recommended as Yes and Yes with modifications [55, 57, 64].

**Table 2:** Overall Recommendations for Use of Appraised Guidelines

	Overall Guideline		CAM Section	
Guideline	Appraiser 1	Appraiser 2	Appraiser 1	Appraiser 2
Piotrowski 2017 [52]	No	No	No	No
Parikh 2016 [53]	Yes with modifications	Yes with modifications	Yes with modifications	Yes with modifications
Ravindran 2016 [54]	Yes with modifications	Yes with modifications	Yes with modifications	Yes with modifications
Oslin 2016 [55]	Yes with modifications	Yes	Yes with modifications	Yes with modifications
Jobst 2016 [56]	Yes with modifications	Yes with modifications	No	No
Trangle 2016 [57]	Yes with modifications	Yes	Yes with modifications	Yes with modifications
Zhao 2015 [58]	Yes with modifications	Yes with modifications	Yes with modifications	Yes with modifications
Cleare 2015 [59]	Yes with modifications	Yes with modifications	No	Yes with modifications
Bauer 2015 [60]	Yes with modifications	Yes with modifications	No	No
Álvarez-Ariza 2014 [61]	Yes with modifications	Yes with modifications	Yes with modifications	Yes with modifications
Bauer 2013 [62]	Yes with modifications	Yes with modifications	Yes with modifications	Yes with modifications
Schwenk 2011 [63]	Yes with modifications	Yes with modifications	No	No
Gelenberg 2010 [64]	Yes with modifications	Yes	Yes with modifications	No
NCCMH 2010 [65]	Yes	Yes	Yes	Yes
Henderson 2010 [66]	Yes with modifications	Yes with modifications	Yes with modifications	Yes with modifications
Malhi 2009 [67]	Yes with modifications	Yes with modifications	No	No

### Overall recommendations: CAM sections (Table 2)

The two appraisers only recommended 1 of the 16 guidelines as Yes without modifications [65]. For 13 of the 16 guidelines, both appraisers concurred in their decision regarding the overall recommendation; five guidelines were rated as No by both appraisers [52, 56, 60, 63, 67] and 8 were rated as Yes with modifications by both appraisers [53–55, 57, 58, 61, 62, 66]. Finally, 2 guidelines were recommended by the two appraisers as No and Yes with modifications [59, 64].

### Scaled domain percentage quality assessment (Table 3)

The scaled domain percentages of the overall guidelines were generated: for the scope and purpose domain, the lowest score was 41.7%, while the highest score was 100.0%; for the stakeholder involvement domain, the lowest score was 36.1%, while the highest was 88.9%; for the rigor-of-development domain, the lowest score was 20.8%, while the highest was 83.3%, for the clarity-of-presentation domain, the lowest score was 75.0%, while the highest was 100.0%; for the applicability domain, the lowest score was 8.3%, while the highest was 72.9%; and for the editorial independence domain, the lowest score was 25.0%, while the highest was 95.8%.

**Table 3:** Scaled Domain Percentages for Appraisers of Each Guideline

Guideline		Domain score (%)					
		Scope and purpose	Stakeholder involvement	Rigour of development	Clarity of presentation	Applicability	Editorial Independence
Piotrowski 2017 [52]	Overall Guideline	41.7	44.4	20.8	75.0	33.3	68.8
	CAM Section	33.3	8.3	4.2	33.3	4.2	68.8
Parikh 2016 [53]	Overall Guideline	86.1	58.3	61.5	75.0	22.9	62.5
	CAM Section	86.1	30.6	55.2	75.0	20.8	62.5
Ravindran 2016 [54]	Overall Guideline	88.9	55.6	60.4	75.0	27.1	62.5
	CAM Section	88.9	58.3	58.3	75.0	27.1	62.5
Oslin 2016 [55]	Overall Guideline	94.4	83.3	82.3	94.4	64.6	25.0
	CAM Section	94.4	77.8	71.9	86.1	41.7	25.0
Jobst 2016 [56]	Overall Guideline	63.9	47.2	62.5	80.6	8.3	95.8
	CAM Section	63.9	50.0	59.4	22.2	2.1	95.8
Trangle 2016 [57]	Overall Guideline	94.4	77.8	79.2	88.9	70.8	87.5
	CAM Section	83.3	47.2	72.9	47.2	29.2	87.5
Zhao 2015 [58]	Overall Guideline	75.0	55.6	49.0	94.4	18.8	41.7
	CAM Section	75.0	58.3	49.0	94.4	18.8	41.7
Cleare 2015 [59]	Overall Guideline	83.3	80.6	45.8	94.4	33.3	87.5
	CAM Section	83.3	38.9	42.7	91.7	18.8	87.5
Bauer 2015 [60]	Overall Guideline	75.0	58.3	40.6	83.3	27.1	70.8
	CAM Section	61.1	33.3	36.5	13.9	12.5	70.8
Álvarez-Ariza 2014 [61]	Overall Guideline	94.4	88.9	61.5	94.4	68.8	54.2
	CAM Section	94.4	36.1	57.3	80.6	41.7	54.2
Bauer 2013 [62]	Overall Guideline	69.4	61.1	52.1	100.0	31.3	70.8
	CAM Section	69.4	30.6	50.0	100.0	10.4	70.8
Schwenk 2011 [63]	Overall Guideline	80.6	41.7	45.8	80.6	50.0	25.0
	CAM Section	80.6	41.7	37.5	44.4	6.3	25.0
Gelenberg 2010 [64]	Overall Guideline	83.3	36.1	79.2	88.9	62.5	91.7
	CAM Section	75.0	27.8	62.5	72.2	56.3	91.7
NCCMH 2010 [65]	Overall Guideline	100.0	83.3	83.3	97.2	72.9	66.7
	CAM Section	100.0	69.4	83.3	97.2	58.3	66.7
SIGN 2010 [66]	Overall Guideline	94.4	75.0	64.6	77.8	47.9	25.0
	CAM Section	94.4	75.0	59.4	88.9	43.8	25.0
Malhi 2009 [67]	Overall Guideline	69.4	44.4	39.6	91.7	35.4	50.0
	CAM Section	58.3	25.0	31.3	36.1	14.6	50.0

The scaled domain percentages of the CAM sections of the guidelines were also generated: for the scope and purpose domain, the lowest score was 33.3%, while the highest was 100.0%; for the stakeholder involvement domain, the lowest score was 8.3%, while the highest was 77.8%; for the rigor-of-development domain, the lowest score was 4.2%, while the highest was 83.3%; for the clarity-of-presentation domain, the lowest score was 13.9%, while the highest was 100.0%; for the applicability domain, the lowest score was 2.1%, while the highest was 58.3%; and for the editorial independence domain, the lowest score was 25.0%, while the highest was 95.8%.

### Scope and purpose

With regard to the overall scaled scores, the overall objectives and health questions were specific and clear for all the guidelines except one [52]. Moreover, for those guidelines with high overall scaled scores, the purpose and health intent were well defined. Meanwhile, most guidelines had CAM scaled scores that were equal to their overall scaled scores in this domain, with the exception of 4 guidelines [52, 57, 60, 64, 67]. For those guidelines that had the same CAM and overall scaled score, the overall objectives encompassed CAM therapies or did not explicitly exclude them. The guidelines with lower CAM scaled scores generally had vague, if any, health questions specific to CAM as compared to specific health questions listed for the non-CAM therapies [52, 57, 60, 64, 67]. Meanwhile, the target populations were generally well-defined for all guidelines and there was no distinction made between the target population for CAM versus non-CAM therapies.

### Stakeholder involvement

Most guidelines described the guideline development group members well; two guidelines fulfilled all of the criteria including the names, affiliations, expertise and geographical locations of the development members [52, 55], while the remaining were missing at least one of the criteria [53, 54, 56–67]. A few guidelines included CAM experts; some provided the same level of description for CAM experts as the other members [54–56, 58, 63, 66], and one guideline had CAM experts listed as some of the consulted stakeholders, with unclear involvement and little to no description [65]. Only two guidelines described the views and preferences of the target audience in detail [61, 65], while most of the guidelines described them vaguely [52, 55–57, 59, 62, 64, 66]. In terms of the CAM subsections, most guidelines had no difference between their description level of the views and preferences of CAM therapies in the target population compared to the non-CAM therapies, except for two which provided less detail [59, 61]. A few guidelines described both the intended audience and how the guideline may be used [55, 57, 60–62, 65], while the remaining provided a description of either the intended audience or usage, except for one which was missing a description of both [52]. There was no difference between the overall and the CAM scores for the description of the target users as the target users were stated for the entire guideline and not for specific therapies.

### Rigor of development

The majority of the guidelines used well-defined systematic methods to search for evidence for the overall guidelines [53–57, 61, 63–66], while some lacked a comprehensive search strategy [52, 58–60, 62, 67]. Some of the guidelines clearly defined their criteria for selecting the evidence [55–57, 63–65], while the remaining provided minimal detail. In terms of the CAM sections, most guidelines clearly used systematic methods to search for CAM-related evidence except for two [52, 63]. One guideline described the criteria for selecting evidence pertaining to CAM therapies in less detail compared to that of the non-CAM therapies [64]. The strengths and limitations of the overall evidence of almost all of the guidelines were well-defined, while one had a vague description [63], and one lacked a description of the strengths and limitations of the evidence altogether [52]. There was only one guideline that described the strengths and limitations of CAM evidence vaguely relative to the overall evidence [63]. The methods for formulating the recommendations were clearly described for most guidelines [53, 54, 56, 64, 65, 67], and the quality of the CAM sections for this item was the same as the overall guidelines. The health benefits, side effects, risks and trade-offs for the recommendations were described by most guidelines to varying degrees. In regards to the CAM sections, a few guidelines failed to list the benefits or harms for all of the CAM therapies, only listing them for a few [55, 57, 63] and one guideline did not mention any consideration of health benefits, side effects, and risks in formulating CAM recommendations [52]. All the guidelines made an explicit link between the overall recommendations and supporting evidence. There was only one guideline that did not provide an explicit link between the CAM recommendations and the supporting evidence [52], while the remaining guidelines did. Some guidelines vaguely stated the involvement of an external review [55, 57, 61, 64–66], and two guidelines clearly stated that no external review was conducted [53, 54]. None of the guidelines explicitly had CAM experts involved in an external review process, except for one [66]. Most guidelines provided a statement that the guideline will be updated [55, 57, 58, 61, 62, 64–66], with one outlining a detailed methodology for the updating procedure. The CAM scores were the same as the overall scores for the updating procedure.

### Clarity of presentation

The overall recommendations of all guidelines were specific and unambiguous, except for one [66]. On the other hand, the CAM recommendations for many guidelines were vague or had limited detail [52, 55–57, 60, 61, 63, 67]. All guidelines provided the different options of non-CAM therapies for the management and/or treatment of the depression clearly. In contrast, some guidelines presented limited or vague CAM therapy options within the scope of the guideline [52, 56, 60, 63, 67]. The overall key recommendations were easily identifiable for many guidelines [52, 55–59, 61–67]. Some guidelines did not present their CAM recommendations clearly [52–54, 56, 57, 60, 63, 67].

### Applicability

One guideline described facilitators and barriers for the implementation of both the overall and the CAM-specific recommendations in detail [64], while some failed to address the facilitators and barriers clearly for both sections [54, 56, 60, 62]. The remaining guidelines vaguely addressed these factors for both sections, with a few guidelines simply listing some factors that applied to all treatment modalities for their CAM sections [52, 53, 55, 57–59, 61, 63, 65–67]. The majority of the guidelines provided advice and/or tools to facilitate the integration of the overall recommendations into practice except for two [56, 59]. There were some guidelines that did not provide any advice or tools specifically for CAM recommendations [52, 56, 59, 62, 63]; two guidelines only provided an implementation section that addressed advice for all treatments in general thereby encompassing CAM recommendations [58, 60]. A few guidelines described the resource implications of implementing the overall recommendations [53, 55, 57, 59, 61–66]. In regards to the CAM sections, two guidelines sufficiently reported the resource implications for CAM recommendations [55, 66], while some guidelines addressed the implications vaguely with generalized statements applying to all modalities [53, 57, 62, 64, 66]. Some guidelines vaguely described monitoring or auditing criteria for the overall sections [53, 54, 56, 58, 63, 66, 67], while the others described it more clearly. Some guidelines did not adequately report any monitoring criteria for CAM-specific recommendations [52, 53, 55, 56, 58, 60, 63] and one guideline reported the criteria thoroughly for both the overall and CAM sections [64].

### Editorial Independence

Some guidelines did not explicitly state that the funding body did not influence the content of guidelines [52–54, 58, 61, 65–67], and two guidelines disclosed the influence of the funding body in place of that statement [57, 64]. Two guidelines simply stated that there was no “commercial funding body” involved in the guideline development [60, 62], and two guidelines did not comment on funding [55, 63]. Competing interests of the guideline development members were addressed and reported in the majority of the guidelines, with most of them failing to report the methods used to seek competing interests, their potential influence on the development process, and/or the types of competing interests considered [53–56, 58–67], except for one guideline that sufficiently reported all the criteria [57], and one guideline which simply disclosed a statement that there were no competing interests amongst the development group members [52].

## Discussion

The aim of this study was to identify the quantity and assess the quality of CAM recommendations in guidelines for the treatment and/or management of depression in adults. Evidence-based clinical practice guidelines were identified to support communication and decision-making between patients and healthcare providers regarding CAM use for depression. Based on our systematic search, we did not find any prior studies in the literature with the same scope and purpose as the present study. This review identified a total of 19 depression guidelines published between 2008 and 2018; of these, 17 mentioned CAM and 16 provided CAM therapy recommendations. The 16 guidelines with CAM recommendations were appraised using the 23-item AGREE II instrument that assessed and scored content and format of guidelines across 6 domains; there was high variability of scores, and thus quality, between overall guidelines and between domains of individual guidelines. In addition, it was found that overall recommendations were generally of higher quality than the CAM recommendations.

In terms of the appraisal of the overall guidelines, 10 guidelines achieved or exceeded a score of 4.5 in both average appraisal score and average overall assessment [53–55, 57, 59, 61, 62, 64–66], 5 guidelines achieved or exceeded a score of 5.0 in both metrics [55, 57, 61, 64, 65], and 1 guideline achieved a score less than 4.0 in both of these metrics [52]. In terms of the appraisal of the CAM sections of the guidelines, 11 guidelines achieved or exceeded a score of 4.0 in average appraisal score and average overall assessment [53–55, 57–59, 61, 62, 64–66], 2 guidelines achieved or exceeded a score of 5.0 in both metrics [55, 65], 2 guidelines achieved a score of 3.0 or less in both metrics [52, 67], and 1 guideline achieved a score lower than 2.0 in both metrics [52] (1 = strongly disagree; 7 = strongly agree that criteria are met). The scaled domain percentages of the overall and CAM sections of the 16 guidelines with CAM recommendations were generated. In regards to the average scaled domain percentages of the overall guidelines, the domain that scored the highest was clarity of presentation at 87.0%, followed by scope and purpose at 80.9%, stakeholder involvement at 62.0%, editorial independence at 61.6%, rigour of development at 58.0%, and the domain that scored the lowest was applicability at 42.2%. In regards to the average scaled domain percentages of the CAM sections of the guidelines, the domain that scored the highest was scope and purpose at 77.6%, followed by clarity of presentation at 66.1%, editorial independence at 61.6%, rigour of development at 52.0%, stakeholder involvement at 44.3%, and the domain that scored the lowest was applicability at 25.4%. Although there are variances in the scaled scores for the overall guidelines versus the scaled sores for the CAM sections of the guidelines, the order of domain quality is similar between the two. In addition, the CAM scaled percentages were lower than the overall scaled percentages for all domains except for editorial independence.

Guidelines that had higher AGREE II scores in overall sections also tended to have higher scores in their CAM sections. Generally, characteristics of guidelines, including the year of publication and region of development varied across the guidelines irrespective of quality. The two guidelines that had the highest scores in both the overall and the CAM sections were published in the USA [55] or the UK [65], and the guidelines that scored the lowest in both their overall and CAM sections were published in Australia [67] and Poland [52]. Both the oldest guideline [67] and the most recent guideline [52] had the lowest scores.

The guideline that scored highest in both overall and CAM sections recommended exercise, St. John’s Wort, mindfulness-based cognitive therapy, bibliotherapy, acceptance and commitment therapy (ACT) for the treatment of depression, and recommended against using omega-3 fatty acids and vitamin D [55] (Fig. 2). This guideline also concluded that there is insufficient evidence to make recommendations regarding the use of acupuncture, yoga, tai chi, or qi gong for the treatment of depression [55].

CAM therapies in the guidelines were referenced using different terminology; ten did not refer to them as CAM [52, 53, 56, 58, 60–63, 65, 67], 3 guidelines referred to them as “complementary and alternative” [54, 64, 66], 1 referred to them as “integrative” [57], 1 referred to them as “complementary and alternative” and “complementary and integrative” [55], and 1 referred to them as “complementary and other treatments” [59].

All the guidelines that referred to CAM using one of the aforementioned terms, except for the one that entirely focused on CAM therapies [54], did not refer to some CAM therapies such as exercise/physical activity, (guided) self-help/self-management, bibliotherapy, herbal and nutritional supplements, light therapy, and/or mindfulness-based therapy as CAM or within the CAM sections of the guidelines [55, 57, 59, 64, 66].

Our findings are comparable to published studies which assessed guideline quality on similar topics. One study appraised 25 guidelines for depression and anxiety in children or youth using the AGREE II instrument [27]. They determined that the frequency of overall guidelines that achieved high scores in each domain, from greatest to least, was as follows: clarity of presentation, scope and purpose, stake holder involvement, editorial independence, rigour of development and finally, applicability [27], aligning with the findings of this study. In addition, previous literature appraising overall and CAM sections of guidelines of various conditions found that the overall scaled domain scores were generally higher than the CAM scaled domain scores, which is also consistent with the results of this study [71–75]. One such study that appraised 17 guidelines on low back pain for both overall and CAM sections, determined that the highest ranking domain was scope and purpose (88.6% overall, 87.1% CAM), followed by clarity of presentation (83.0% overall, 73.2% CAM), and the lowest ranking domain was applicability (31.8, 21.8%), similar to this study’s findings [72]. Likewise, other similar studies appraising guidelines of various conditions [25–27, 71–79], including the recent study that appraised 11 guidelines on depression in adults [25], had results similar to this review, demonstrating that the variable and sub-optimal quality of guidelines is consistent with the literature and is not unique.

Moreover, this study revealed that several guidelines containing CAM recommendations for the treatment and/or management of depression in adults are available to support informed and shared decision-making between patients and healthcare providers. The total number of CAM therapies that were recommended, as well as the number of guidelines containing CAM recommendations (*n* = 16), were generally considerably greater than those reported in similar reviews appraising guidelines of other conditions [71–75]. It was determined that the quality of CAM recommendations was lower than the overall recommendations in the guidelines despite the growing and high prevalence of CAM use for depression [8–15]. This study thereby also highlighted the need for more research supporting the development of higher quality evidence-based CAM-containing guidelines for depression.

### Strengths and limitations

Strengths of this study include the use of a comprehensive systematic review to identify eligible guidelines for the treatment and/or management of depression, as well as the use of the AGREE II instrument, a valid and well-accepted appraisal instrument for guideline quality assessment [30]. Limitations include the fact that only two appraisers assessed guidelines using AGREE II, as opposed to four appraisers as recommended by the instrument’s user manual. To mitigate this and standardize scoring, all three authors independently appraised three guidelines in an initial pilot test and discussed the results and application of the instrument. Furthermore, following the appraisal of all guidelines assessed for this study, any discrepancies were discussed, and scores were carefully reviewed. Finally, this study did not capture any guidelines published outside of the English language, and thus does not account for guidelines that may capture recommendations for traditional medicines or CAM endemic to non-English speaking regions of the world.

## Conclusions

This study identified 16 guidelines published between 2008 and 2018 providing CAM recommendations for the treatment and/or management of depression in adults. Appraisal of these guidelines using the AGREE II instrument demonstrated that quality varied within and across guidelines and quality of CAM recommendations was generally lower than the overall recommendations of the guidelines. This study also revealed that there are multiple depression guidelines containing CAM recommendations available and there are a comprehensive set of CAM therapy options for depression. The guidelines with higher AGREE II scores and favourable overall recommendations can serve as a guide to facilitate communication between patients and healthcare providers regarding the use of CAM therapies in the treatment and/or management of depression. Guidelines that scored variable or lower scaled domain percentages and unfavourable overall recommendations could be improved in future updates, using the AGREE II instrument (among other guideline development tools) as a guide.

## Supplementary information

**Additional file 1: Supplementary File 1.** MEDLINE Search Strategy for Depression Clinical Practice Guidelines Executed Oct 11, 2018.

**Additional file 2: Supplementary File 2.** Modified AGREE II Questions Used to Guide Scoring of CAM Sections of Each Guideline.

**Additional file 3: Supplementary File 3.** Average Appraisal Scores, Average Overall Assessments and Recommendations Regarding Use of Guidelines.

## Data Availability

All relevant data are included in this manuscript.

## Abbreviations

AGREE II: Appraisal of Guidelines for Research & Evaluation II
CAM: Complementary and Alternative Medicine
NCCIH: National Center for Complementary and Integrative Health
PICO: Patients, Intervention, Comparison and Outcomes
PRISMA: Preferred Reporting Items for Systematic Reviews and Meta-Analyses
