# Supplementary material for: Complementary and alternative medicine recommendations for depression: a systematic review and assessment of clinical practice guidelines
Source: BMC Complement Med Ther. 2020 Oct 7;20:299. doi: 10.1186/s12906-020-03085-1 (PMC7541317; doi:10.1186/s12906-020-03085-1)
Supplement: Supplementary file 1 — Additional file 1: Supplementary File 1. MEDLINE Search Strategy for Depression Clinical Practice Guidelines Executed Oct 11, 2018. [file 12906_2020_3085_MOESM1_ESM.docx]

## Supplementary File 1: MEDLINE Search Strategy for Depression Clinical Practice Guidelines Executed Oct 11, 2018

| Database: Ovid MEDLINE(R) and Epub Ahead of Print, In-Process & Other Non-Indexed Citations, Daily and Versions(R) <1946 to October 09, 2018>  Search Strategy:  --------------------------------------------------------------------------------  1 depression.mp. or DEPRESSION/ (360679)  2 major depressive disorder.mp. or Depressive Disorder, Major/ (35823)  3 depressive disorder.mp. or Depressive Disorder/ (103307)  4 or/1-3 (392053)  5 limit 4 to ("all infant (birth to 23 months)" or "all child (0 to 18 years)" or "newborn infant (birth to 1 month)" or "infant (1 to 23 months)" or "preschool child (2 to 5 years)" or "child (6 to 12 years)" or "adolescent (13 to 18 years)") (67135)  6 4 not 5 (324918)  7 limit 6 to (english language and humans and yr="2008 -2018" and (guideline or practice guideline)) (96)  *************************** |
| --- |

## 
