# Supplementary material for: Complementary and alternative medicine recommendations for depression: a systematic review and assessment of clinical practice guidelines
Source: BMC Complement Med Ther. 2020 Oct 7;20:299. doi: 10.1186/s12906-020-03085-1 (PMC7541317; doi:10.1186/s12906-020-03085-1)
Supplement: Supplementary file 3 — Additional file 3: Supplementary File 3. Average Appraisal Scores, Average Overall Assessments and Recommendations Regarding Use of Guidelines. [file 12906_2020_3085_MOESM3_ESM.docx]

## Supplementary File 3: Average appraisal scores and average overall assessments of each guideline

| Guideline | Metric | Appraiser 1 | Appraiser 2 | Average | Standard Deviation |
| --- | --- | --- | --- | --- | --- |
| Piotrowski 2017 [52]  (Overall) | Appraisal Score | 3.3 | 3.5 | 3.4 | 0.1 |
|  | Overall Assessment | 3.0 | 3.5 | 3.3 | 0.4 |
| Piotrowski 2017 [52]  (CAM Section) | Appraisal Score | 1.6 | 2.3 | 1.9 | 0.5 |
|  | Overall Assessment | 2.0 | 2.0 | 2.0 | 0.0 |
| Parikh 2016 [53]  (Overall) | Appraisal Score | 4.3 | 4.8 | 4.6 | 0.3 |
|  | Overall Assessment | 4.0 | 5.0 | 4.5 | 0.7 |
| Parikh 2016 [53]  (CAM Section) | Appraisal Score | 3.9 | 4.3 | 4.1 | 0.3 |
|  | Overall Assessment | 4.0 | 4.5 | 4.3 | 0.4 |
| Ravindran 2016 [54]  (Overall) | Appraisal Score | 4.4 | 4.8 | 4.6 | 0.3 |
|  | Overall Assessment | 4.0 | 5.0 | 4.5 | 0.7 |
| Ravindran 2016 [54]  (CAM Section) | Appraisal Score | 4.2 | 4.7 | 4.5 | 0.4 |
|  | Overall Assessment | 4.0 | 4.5 | 4.3 | 0.4 |
| Oslin 2016 [55]  (Overall) | Appraisal Score | 5.3 | 6.0 | 5.7 | 0.5 |
|  | Overall Assessment | 5.0 | 6.0 | 5.5 | 0.7 |
| Oslin 2016 [55]  (CAM Section) | Appraisal Score | 5.2 | 5.2 | 5.2 | 0.0 |
|  | Overall Assessment | 5.0 | 5.0 | 5.0 | 0.0 |
| Jobst 2016 [56]  (Overall) | Appraisal Score | 4.4 | 4.3 | 4.4 | 0.1 |
|  | Overall Assessment | 5.0 | 4.5 | 4.8 | 0.4 |
| Jobst 2016 [56]  (CAM Section) | Appraisal Score | 3.6 | 3.8 | 3.7 | 0.1 |
|  | Overall Assessment | 3.0 | 3.0 | 3.0 | 0.0 |
| Trangle 2016 [57]  (Overall) | Appraisal Score | 5.6 | 6.2 | 5.9 | 0.4 |
|  | Overall Assessment | 5.0 | 6.0 | 5.5 | 0.7 |
| Trangle 2016 [57]  (CAM Section) | Appraisal Score | 4.5 | 4.7 | 4.6 | 0.1 |
|  | Overall Assessment | 4.0 | 4.5 | 4.3 | 0.4 |
| Zhao 2015 [58]  (Overall) | Appraisal Score | 4.1 | 4.3 | 4.2 | 0.1 |
|  | Overall Assessment | 4.0 | 4.0 | 4.0 | 0.0 |
| Zhao 2015 [58]  (CAM Section) | Appraisal Score | 4.1 | 4.3 | 4.2 | 0.1 |
|  | Overall Assessment | 4.0 | 4.0 | 4.0 | 0.0 |
| Cleare 2015 [59]  (Overall) | Appraisal Score | 4.7 | 4.8 | 4.8 | 0.1 |
|  | Overall Assessment | 4.0 | 5.0 | 4.5 | 0.7 |
| Cleare 2015 [59]  (CAM Section) | Appraisal Score | 3.9 | 4.3 | 4.1 | 0.3 |
|  | Overall Assessment | 4.0 | 4.0 | 4.0 | 0.0 |
| Bauer 2015 [60]  (Overall) | Appraisal Score | 4.0 | 4.4 | 4.2 | 0.3 |
|  | Overall Assessment | 4 | 4 | 4.0 | 0.0 |
| Bauer 2015 [60]  (CAM Section) | Appraisal Score | 2.5 | 3.3 | 2.9 | 0.6 |
|  | Overall Assessment | 3.0 | 3.5 | 3.3 | 0.4 |
| Álvarez Ariza 2014 [61]  (Overall) | Appraisal Score | 5.4 | 5.5 | 5.5 | 0.1 |
|  | Overall Assessment | 5 | 5.5 | 5.3 | 0.4 |
| Álvarez Ariza 2014 [61]  (CAM Section) | Appraisal Score | 4.5 | 4.5 | 4.5 | 0.0 |
|  | Overall Assessment | 4.0 | 4.5 | 4.3 | 0.4 |
| Bauer 2013 [62]  (Overall) | Appraisal Score | 4.5 | 4.7 | 4.6 | 0.2 |
|  | Overall Assessment | 4 | 5 | 4.5 | 0.7 |
| Bauer 2013 [62]  (CAM Section) | Appraisal Score | 3.9 | 4.2 | 4.0 | 0.2 |
|  | Overall Assessment | 4.0 | 4.0 | 4.0 | 0.0 |
| Schwenk 2011 [63]  (Overall) | Appraisal Score | 4.0 | 4.4 | 4.2 | 0.3 |
|  | Overall Assessment | 4.0 | 4.5 | 4.3 | 0.4 |
| Schwenk 2011 [63]  (CAM Section) | Appraisal Score | 3.2 | 3.4 | 3.3 | 0.1 |
|  | Overall Assessment | 3.0 | 3.0 | 3.0 | 0.0 |
| Gelenberg 2010  [64]  (Overall) | Appraisal Score | 5.3 | 5.6 | 5.4 | 0.2 |
|  | Overall Assessment | 5.0 | 6.0 | 5.5 | 0.7 |
| Gelenberg 2010  [64]  (CAM Section) | Appraisal Score | 4.5 | 4.7 | 4.6 | 0.1 |
|  | Overall Assessment | 4.0 | 4.5 | 4.3 | 0.4 |
| NCCMH 2010 [65]  (Overall) | Appraisal Score | 6.0 | 6.1 | 6.0 | 0.1 |
|  | Overall Assessment | 6.0 | 6.0 | 6.0 | 0.0 |
| NCCMH 2010 [65]  (CAM Section) | Appraisal Score | 5.7 | 5.9 | 5.8 | 0.1 |
|  | Overall Assessment | 6.0 | 6.0 | 6.0 | 0.0 |
| Henderson 2010 [66]  (Overall) | Appraisal Score | 4.8 | 5.0 | 4.9 | 0.2 |
|  | Overall Assessment | 4.0 | 5.0 | 4.5 | 0.7 |
| Henderson 2010 [66]  (CAM Section) | Appraisal Score | 4.7 | 5.0 | 4.8 | 0.2 |
|  | Overall Assessment | 4.0 | 5.0 | 4.5 | 0.7 |
| Malhi 2009 [67]  (Overall) | Appraisal Score | 4.0 | 4.2 | 4.1 | 0.2 |
|  | Overall Assessment | 4 | 4 | 4.0 | 0.0 |
| Malhi 2009 [67]  (CAM Section) | Appraisal Score | 2.8 | 2.9 | 2.8 | 0.1 |
|  | Overall Assessment | 3.0 | 3.0 | 3.0 | 0.0 |
